# Risk and Protective Factors for Intimate Partner Violence Against Bisexual Victims: A Systematic Scoping Review

**DOI:** 10.1177/15248380221084749

**Published:** 2022-04-17

**Authors:** Julia Corey, Marian Duggan, Áine Travers

**Affiliations:** 1 Trinity Centre for Global Health, School of Psychology, 8809 Trinity College Dublin, College Green, Dublin 2, Ireland; 2 School of Social Policy, Sociology and Social Research, 2240 University of Kent, Canterbury, UK

**Keywords:** intimate partner violence, partner abuse, domestic abuse, bisexuality, LGBTQ, scoping review

## Abstract

Bisexual-identifying individuals appear to be at increased risk of experiencing intimate partner violence (IPV) compared to people of other sexualities. The purpose of this systematic scoping review was to examine risk and protective factors for the perpetration of IPV against bisexual victims and to provide a preliminary quality assessment of the included studies. A systematic search of academic and grey literature was conducted in February 2021. Inclusion criteria specified that study participants identified as bisexual, that the study examined risk or protective factors for IPV, and that findings were disaggregated by sexual identity. All potentially eligible references were independently screened by two reviewers, and conflicts settled by a third reviewer. Nine articles published between 2013 and 2021 met criteria for inclusion. Data extraction was completed for all included studies, and findings presented in a narrative synthesis. The review identified a number of risk factors, including bisexual identity, internalised homophobia, discrimination, partner gender, negative childhood experiences and non-monogamy. One study included consideration of a potentially protective factor. The majority of the included studies were cross-sectional in design. More longitudinal studies are needed to clarify temporality of the associations identified and better inform support and prevention efforts. Further implications for future research, policies and practise are discussed.

## Introduction

Intimate partner violence (IPV) refers to physical, emotional or psychological abuse perpetrated by a current or former partner. IPV is a major health and human rights concern worldwide (Ellsberg et al., 2008; Garcia-Moreno et al., 2006). The World Health Organization (WHO) estimates that nearly one third of women who have been in a relationship have experienced physical or sexual violence by an intimate partner (WHO, 2013). IPV affects every demographic, including members of the lesbian, gay, bisexual, transgender, queer, plus (LGBTQ+) community. Although the majority of research focuses on IPV experienced by women within heterosexual relationships, research suggests that lesbian, gay and bisexual individuals may experience IPV at similar or higher rates than their heterosexual counterparts (Walters et al., 2013), and may face additional barriers to help seeking (Calton et al., 2016).

A 2013 survey from the US Centers for Disease Control and Prevention (CDC) suggested that bisexual individuals reported higher rates of IPV than their heterosexual, gay or lesbian counterparts. The CDC data showed that more than 61% of bisexual women and 37% of bisexual men reported experiencing intimate partner rape, physical violence or stalking, compared with 44% of lesbians, 35% of heterosexual women, 26% of gay men and 29% of heterosexual men (Walters et al., 2013). Additionally, a report by the U.K. Office for National Statistics (ONS) found that bisexual women in England and Wales were almost twice as likely to report past year IPV than heterosexual women (11% compared with 6%, respectively) (ONS, 2018). When broken down by abuse type, bisexual women were nearly five times more likely to have experienced sexual violence by a current or former intimate partner (ONS, 2018).

As well as higher risk of experiencing IPV, bisexual individuals also appear to receive lower social support and have poorer mental health than their monosexual peers. Bisexual individuals are exposed to minority stress, which refers to the chronic stress that arises from being identified as a stigmatised minority group and associated experiences of discrimination and prejudice, expectations of rejection, concealment of identity and internalised stigma (Meyer, 1995, 2003). It may be the case that bisexual people experience greater levels of minority stress than other sexual minority groups, due to reported experiences of ostracisation from both heterosexual and lesbian/gay communities (Hayfield et al., 2014). A CDC report found that bisexual individuals experienced serious psychological distress at nearly twice the rate of heterosexual participants (Ward et al., 2014) These findings suggest that bisexual individuals may also experience worse outcomes after victimisation. Indeed, a study by Dickerson-Amaya and Coston (2019) reported that bisexual men were more likely to report poor mental health after experiencing IPV than gay and heterosexual men. This suggests that individuals identifying as bisexual are a particularly high-risk group with respect to IPV. However, for research purposes, LGBTQ+ individuals are commonly treated as one homogenous group.

The practice of treating LGBTQ+ individuals as one group may obscure specific risks facing each individual sexual or gender minority. Bisexual victims may be less visible in the context of services and support for victims of IPV, where they may be more likely to be viewed as either heterosexual or gay, depending on whether they are currently in a same-sex or opposite-sex relationship. This may further obfuscate sexuality-specific risk or protective factors relevant to prevention of and intervention for IPV. The increased risk of IPV among LGBTQ+ individuals has been attributed to the presence of minority stress (Decker et al., 2018). However, minority stress theory alone does not explain why risk would be elevated among bisexual victims compared with those identifying as lesbian or gay.

Enhanced understanding of risk and protective factors is therefore required to inform the development of effective prevention and intervention strategies and programmes. A recent review by Bermea et al. (2018) examined IPV among bisexual women and included a broad discussion of prevalence and correlates of IPV affecting this group. This review builds upon the work of Bermea et al. (2018) by examining factors relating to victims of all genders, including a more detailed and systematic examination of empirically identified risk and protective factors, and providing a preliminary quality assessment of the included studies. Therefore, the primary aim of this review is to identify risk and protective factors for IPV among bisexual-identifying individuals, and its research question is: which factors are associated with increased or decreased risk of experiencing IPV among bisexual-identified individuals?

## Methodology

### Search Strategy

A systematic search was conducted on five databases (Web of Science, EMBASE, PubMed, PsycINFO and SCOPUS), selected with the intention of including a wide array of sources from the health and social sciences. The databases were searched using the following search terms: bisexual, pansexual, queer, non-monosexual, sexual minority, LGB*, men who have sex with men, MSM, women who have sex with women, WSW, non-heterosexual, diverse sexualities, same-sex, sexual and gender minorities, SGM. These terms were cross-referenced with the terms partner violence, intimate partner violence, IPV, domestic violence, spousal violence, partner abuse, spouse abuse, dating abuse, domestic abuse, dating violence and family violence. Reference lists of relevant studies and other review articles (Bermea et al., 2018; Johnson & Grove, 2017; Turell et al., 2018; Vencill & Israel, 2018) on similar topics were also examined to identify additional articles. Corresponding authors of relevant studies were emailed to identify potentially relevant additional research. Finally, 14 websites of relevant LGBTQ+ advocacy and service provider organisations (CDC, Guttmacher Institute, Human Rights Campaign, Stonewall, Safelives, LGBT Foundation, This is Biscuit, Survivors’ Network, Biphoria, LGBT MAP, National LGBTQ Task Force, Office for National Statistics [UK], Gov.uk and Consortium.lgbt), were searched to identify potentially relevant research published as reports.

### Study Selection

The literature search was conducted on 12th February 2021. Identified articles were exported first to an EndNote library, and then uploaded to the systematic review screening platform Covidence (www.covidence.org) for removal of duplicates and screening. Articles were independently screened by the first and last authors, and minor disagreements in the rating of articles were resolved through discussion and with the input of the second author. The inclusion criteria applied for the present review selected articles that (1) included quantitative or qualitative analysis of risk or protective factors for IPV perpetration against adult (18+) individuals identifying as bisexual; (2) were written in English; and (3) published in a peer-reviewed journal, or as a book chapter or a technical report. Studies were excluded if they were (1) not primary research; (2) dissertations or unpublished; or (3) included individuals under the age of 18 in analysis. It was not a requirement for the included studies to focus solely on bisexual individuals, but it was required that they provide data disaggregated by sexual orientation, to allow for assessment of risk or protective factors experienced specifically by bisexual people.

The decision to screen reports published, for example, by advocacy organisations, but not dissertations and other unpublished material, represents an attempt to strike a balance between performing a wide and inclusive search, yet also establishing some minimum standard of rigour and quality control for the included material. In this case, publication of some sort was taken to represent this minimum required standard, although it is acknowledged that this may have resulted in the exclusion of some potentially relevant material.

### Data Charting and Synthesis

Data from included studies were first extracted using a table specifically designed by the research team to capture pre-specified aspects of study design (cross-sectional/longitudinal and quantitative/qualitative), sample characteristics (genders, age, sampling strategy and sexual identities) and results. A condensed version of this extraction is presented in Table 1. Findings were then analysed in a narrative synthesis (Mays et al., 2005), presented in the following section. A tabular and narrative synthesis provides a means of discussing and interpreting findings and characteristics of methodologically diverse studies, and is therefore considered appropriate for the chosen design of this review (a systematic scoping review; Grant & Booth, 2009).

**Table 1.** Study characteristics, main findings and risk factors identified.

Authors (year), country	Design	Sample characteristics	Main findings	Risk factors identified
Coston (2017), US	Cross-sectional, quantitative	Women, 18+ (*n* = 3076). Sampled as part of wider survey on IPV, using random digit dialling. Heterosexual/straight (*n* = 2,423), lesbian(*n* = 76), bisexual (*n* = 160), behaviourally non-monosexual women identifying as straight or lesbian, or non-labelled(*n* = 417)	Non-monosexual women have increased risk of experiencing sexual, emotional, and psychological violence and stalking from intimate partners, but equivalent risk of physical violence	Social power
				Women were at increased risk for IPV if partner was a man, but this risk became non-significant for some types of violence when controlling for cumulative inequality and sexual identity Self-identifying as bisexual
Finneran & Stephenson (2014), US	Cross-sectional, quantitative	Men, 18–50+ (*n* = 1575). Sampled via social media advertisements. Homosexual (*n* = 1,453), bisexual (*n* = 122)	Minority stress, including from racism, internalised homophobia, and homophobic discrimination, increases risk for experiencing IPV. Internalised homophobia and homophobic discrimination significantly associated with sexual violence perpetration	Self-identifying bisexual men had higher internalised homophobia and reported more racial discrimination than homosexual men
Goldberg & Meyer (2013), US	Cross-sectional, quantitative	Men and women, 18–70 (*n* = 31,623). Part of health survey. Sampled via random dialling, stratified by counties Heterosexual women(*n* = 16,926), lesbian women (*n* = 267), bisexual women(*n* = 247), WSW (*n* = 94) Heterosexual men (*n* = 13,447) Gay men (*n* = 415), bisexual men (*n* = 135), MSM (*n* = 92)	Prevalence of lifetime and 1-year IPV was significantly higher among bisexual women and gay men than heterosexuals. Psychological distress and binge drinking did not explain the higher prevalence rates	Male perpetrators accounted for 95% of most recent 1-year IPV against bisexual women
Head & Milton, (2014), UK	Cross-sectional, qualitative	Bisexual men (*n* = 2) and women (*n* = 8), age 21–49 (M = 31). Sampled via LGBT organisations, social media networks, and academic mailing lists Bisexual-identifying	The experience of bisexual intimate partner abuse is theorised to be represented by a basic psychological process referred to as ‘adjusting for consonance’ that involves ‘getting lost in the relationship’ (not identifying it as abuse), and ‘lifting the veil’ (beginning to identify abuse and transition out of the relationship)	Lack of representation of healthy bisexual relationships in popular culture Fear of contributing to biphobic societal attitudes if abuse is discussed Engagement in closed or open polyamorous relationships – sometimes provided abusive partners with additional means of exerting control Partner biphobia was a risk Victim exposure to childhood abuse increased risk Insight from experiencing abuse in previous relationship cited as having possible protective effect
Martin-storey and Fromme (2021), US	Longitudinal (four years), quantitative	College students (*n* = 2,474), men and women, mean age = 21.88. College students recruited via summer orientation and by mail. Non college sample recruited online Heterosexual (*n* = 2,342), bisexual (*n* = 63), Gay/Lesbian (*n* = 69)	Sexual minorities had higher rates of dating violence, but association between identity and victimisation became non-significant in the final model. Discrimination mediated association between bisexual identity and dating violence. Sex, number of partners, alcohol use, and childhood maltreatment were associated with dating violence but did not explain increased vulnerability of sexual minorities compared to heterosexual counterparts	Discrimination had a significant indirect effect between bisexual identity and higher rates of dating violence
Pittman et al.(2020), US	Cross-sectional, quantitative	Women, 18–25 (m = 19.73, SD = 1.588). Sampled randomly via nationwide survey of college students Heterosexual (*n* = 7864) Bisexual (*n* = 484) Lesbian (*n* = 108) Asexual (*n* = 667) Pansexual (*n* = 145) Questioning (*n* = 139) Other (*n* = 31)	Regardless of race, sexual minority status increases vulnerability to all forms of IPV (physical, sexual and emotional)	Black bisexual women were at a marginally higher risk for IPV. Bisexual women who slept with a male partner in the past year had significantly higher risk for IPV than any other sexual orientation
Friedman et al. (2019), US	Cross-sectional, quantitative	Men, 18–40+ (*n* = 4430). Sampled from black pride events in six US cities. Gay-identified men who have sex with men only (MSMO; *n* = 3239), gay-identified men who have sex with men and women (MSMW; *n* = 355), heterosexual-identified MSMO (*n* = 13), heterosexual-identified MSMW (*n* = 24), bisexual-identified MSMO (*n* = 370), bisexual-identified MSMW (*n* = 370), other MSMO (*n* = 43), other MSMW (*n*=15), non-responder (*n*=1)	Bisexual and gay-identified MSMW are more likely than gay-identified MSMO to report psychosocial morbidities, including IPV. Lack of gay community support has an indirect effect on the relationship between bisexual behaviour/identity and psychosocial morbidity. There was also an indirect effect of sexuality non-disclosure on the relationship between bisexual behaviour/identity and a lack of community support	Bisexual identity has a significant effect on psychosocial morbidity Non-disclosure of sexuality is associated with less community support. The relationship between bisexuality and psychosocial morbidity is mediated by lack of gay community support
Taylor & Neppl (2020), US	Cross-sectional, quantitative	LGBTQ students (women, men, and gender minorities), age 18–30 (M = 21) (*n* = 379). Sampled via university mailing list Bisexual (45.1%) Gay (17.6%) Lesbian (11.1%) Pansexual (12.2%) Other (14%)	Experiencing microaggressions relating to sexuality was associated with victimisation and perpetration of psychological IPV, when controlling for other variables in the model	Bisexual identity moderated the relationship between psychological victimisation and perpetration from interparental conflict, harsh parenting or microaggressions
Turell et al. (2018), US	Cross-sectional, quantitative	Women, men, transgender, undecided gender, age 18–64, M = 31.53, SD = 9.74 (*n* = 439). Sampled via Facebook and MTurk Bisexual-identifying	Perpetrators’ bi-negativity and real or perceived infidelity were most related to IPV perpetration and victimisation, particularly when the perpetrator was male and both partners were bisexual	Direct effects of (1) both partners being bisexual and (2) partner bi-negativity on victimisation and perpetration Participants’ gender identity (male), and age at time of relationship associated with higher victimisation scores. Partners’ age associated with higher perpetration scores Direct effect of infidelity in relationship on victimisation Direct effect of infidelity and having an open relationship on perpetration scores Involvement in bisexual community associated with higher risk of victimisation Racial identity associated with higher victimisation scores and perpetration scores

A preliminary assessment of study quality is also presented. Given the nature of the research under investigation, and the early stage of inquiry on the topic, a structured quality analysis or risk-of-bias assessment of included articles was not possible. However, the following section includes a preliminary analysis of quality-related characteristics of included studies as a means of producing suggestions for future research and strengthening of the evidence-base in relevant areas.

## Results

### Screening Process

**Figure 1.**
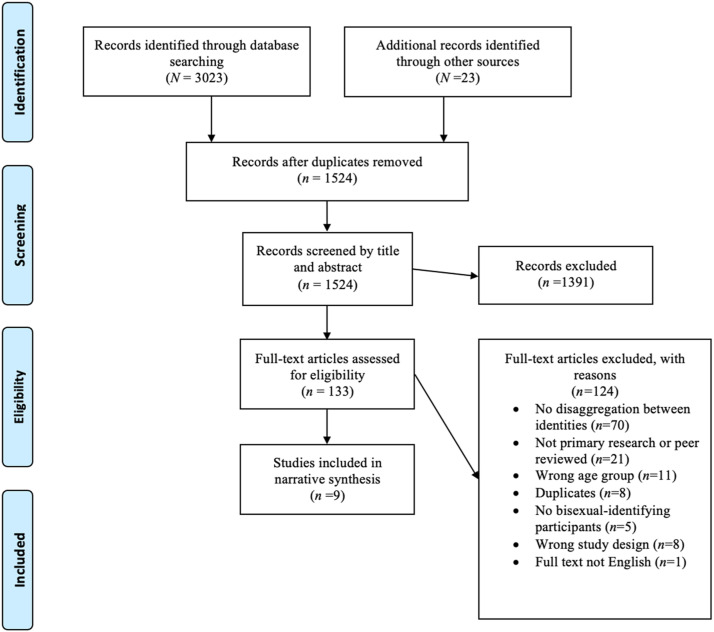
PRISMA Flow Diagram.

As illustrated in Figure 1, the initial search of the five databases returned 3023 articles. Of these, 1522 were identified as duplicate references, leaving a total of 1501 articles identified through the database search for full-text screening. Searching for grey literature via relevant websites, snowballing and emailing corresponding authors yielded an additional 23 references. After screening titles and abstracts, 1391 articles were excluded, and 133 articles remained for full-text screening. Following review of the full-texts, nine articles met the inclusion criteria for analysis.

### Description of Included Studies

Despite evidence that bisexual-identifying individuals are at an increased risk of experiencing IPV, this review identified an overall paucity of literature examining risk and protective factors specific to this group. Few studies examining IPV in LGBTQ+ communities disaggregated findings by sexual identity. Of the nine articles that did assess risk or protective factors for IPV against bisexual victims, eight were quantitative studies, and one was qualitative. Seven articles measured IPV prevalence among multiple sexual identities. The other two examined bisexual-identifying individuals only. All seven of those that examined rates of IPV reported by people of different sexual identities found significantly higher prevalence rates for bisexual victims compared to heterosexual counterparts (Goldberg & Meyer, 2013; Martin-Storey & Fromme, 2021; Pittman et al., 2020), other sexual minorities (Finneran & Stephenson, 2014; Friedman et al., 2019; Taylor & Neppl, 2020) or both (Coston, 2017).

### Preliminary Quality Analysis

All but one study (Martin-Storey & Fromme, 2021) were cross-sectional in design, thus limiting the potential to infer directionality of relationships between the variables investigated. Several included studies were not designed specifically for the purpose of investigating IPV among bisexual individuals; four studies consisted of secondary analysis of data collected as part of broader research projects (Coston, 2017; Friedman et al., 2019; Goldberg & Meyer, 2013; Pittman et al., 2020), while five included studies were designed specifically to examine risk or protective factors for IPV among this group (Finneran & Stephenson, 2014; Head & Milton, 2014; Martin-Storey & Fromme, 2021; Taylor & Neppl, 2020; Turell et al., 2018). Secondary data analysis posed problems in some cases in relation to statistical power. In some cases, even where the overall datasets were large, the numbers of participants identifying as each sexual orientation within them were relatively small. Only one of the included quantitative studies (Taylor & Neppl, 2020) reported a formal power analysis, and the study in question was specifically designed to examine risk factors for IPV in LGBTQ individuals.

Studies differed in their means of measuring IPV occurrence. Five studies measured experiencing or perpetrating IPV dichotomously (yes/no items) (Coston, 2017; Finneran & Stephenson, 2014; Friedman et al., 2019; Goldberg & Meyer, 2013; Pittman et al., 2020), and three used standardised scales. Both Taylor and Neppl (2020) and Martin-Storey and Fromme (2021) used the Revised Conflict Tactics Scale (CTS2) (Straus et al., 2016) to measure IPV victimisation and perpetration while Turell et al. (2018) measured IPV victimisation using the Composite Abuse Scale (CAS) (Hegarty et al., 2005) and perpetration using the Abusive Behavior Inventory (ABI) (Shepard & Campbell, 1992). Additionally, four studies measured exclusively past year IPV (Finneran & Stephenson, 2014; Friedman et al., 2019; Pittman et al., 2020; Taylor & Neppl, 2020), and one study measured only lifetime IPV (Coston, 2017). Goldberg and Meyer (2013) explored both lifetime and one-year IPV, and a longitudinal study by Martin-Storey and Fromme (2021) measured three-month IPV. One study by Turell et al. (2018) asked participants to answer questions pertaining to experiences of IPV in relation to either their current or longest relationship.

Studies further differed in the types of violence they recorded as IPV. Two studies measured emotional, physical and sexual IPV (Coston, 2017; Pittman et al., 2020), with Coston (2017) also measuring partner stalking and control, and one study assessed both physical and sexual IPV (Finneran & Stephenson, 2014). Two studies examined only physical IPV (Friedman et al., 2019; Martin-Storey & Fromme, 2021), and one study assessed only psychological IPV (Valente, 1996). Findings from two articles did not distinguish between different types of IPV, though participants in the study by Goldberg and Meyer (2013) were asked questions pertaining to physical and sexual violence, and Turell et al. (2018) examined severe, emotional, physical and psychological abuse as well as harassment.

### Summary of Risk and Protective Factors Identified

Table 1 presents the characteristics of all included studies, their main findings and the risk or protective factors they identified in relation to the perpetration of IPV against bisexual victims. All of the studies identified at least one risk factor related to IPV victimisation of bisexual-identifying individuals. Four studies examined risk factors for both IPV victimisation and perpetration (Finneran & Stephenson, 2014; Martin-Storey & Fromme, 2021; Taylor & Neppl, 2020; Turell et al., 2018), while five explored only factors related to victimisation (Coston, 2017; Friedman et al., 2019; Goldberg & Meyer, 2013; Head & Milton, 2014; Pittman et al., 2020). Only one study identified a protective factor for IPV victimisation (Head & Milton, 2014). Risk factors for perpetration and victimisation in the following narrative synthesis include factors relating to all levels of the WHO ecological framework – the individual, relational, community and societal levels (Violence and Prevention Alliance, 2021).

#### Bisexual Identity

Findings from three studies indicate that self-identifying as bisexual (as distinct from behavioural bisexuality) may be associated with increased risk of experiencing IPV (Coston, 2017; Friedman et al., 2019; Taylor & Neppl, 2020). Notably, Coston (2017) reported that bisexual-identifying women were significantly more likely to experience all forms of IPV than lesbian and heterosexual-identifying women who were behaviourally bisexual. However, Coston’s (2017) findings were based on a secondary analysis of CDC data, and included relatively small numbers of lesbian-identified participants (*n* = 76), which was further reduced in some of the analyses to as low as *n* = 20. Such low numbers for some identity groups could mean that certain associations may be missed due to low statistical power. A study by Friedman et al. (2019) reported bisexual identity had a significant effect on psychosocial morbidity, measured using a composite construct capturing participants’ experiences of IPV, physical assault, depression symptoms and polydrug use. Bisexual identity was also a significant moderating factor of an association identified between experiencing microaggressions and the perpetration and victimisation of psychological IPV in a study by Taylor and Neppl (2020).

#### Homophobia and Biphobia

Three studies identified relationships between homophobia and experiencing or perpetrating IPV (Finneran & Stephenson, 2014; Head & Milton, 2014; Turell et al., 2018). One study by Finneran and Stephenson (2014) found that self-identifying bisexual men had higher internalised homophobia than homosexual men, and that men who perpetrated sexual violence had higher internalised homophobia than those who did not. Partner bi-negativity, including experiences of hostility, assumptions of promiscuity and sexual irresponsibility, was found to be associated with both IPV victimisation and perpetration in a study by Turell et al. (2018), particularly where the perpetrator was male and both partners were bisexual. Qualitative findings from Head and Milton (2014) also described how partner biphobia contributed to controlling behaviours and breaking sexual boundaries of victims, whereby biphobic stereotypes such as non-monogamy were used by perpetrators as justifications for the abuse.

#### Gender

Three articles (Coston, 2017; Goldberg & Meyer, 2013; Pittman et al., 2020) suggested that bisexual women were at an increased risk of experiencing IPV if their intimate partner was a male. Goldberg and Meyer (2013) reported that 95% of most recent 1-year IPV against bisexual women was perpetrated by male individuals. Pittman et al. (2020) reported in their study discussion that bisexual women who had a male sexual partner in the past year were at a significantly higher risk of IPV than any other sexual orientation, although supporting data for this finding did not appear to be available in the article’s results section. The study by Coston (2017) found partner gender to be a significant factor, such that bisexual women were at increased risk of experiencing stalking and sexual, emotional or psychological abuse at the hands of a male partner than a woman (*OR*s 2.0–6.1). However, Coston (2017) found the effect of partner gender became non-significant for some forms of violence after controlling for a measure of social power (a variable constructed by converting one’s theoretically privileged identity aspects into numerical scores, for example, white vs non-white would be coded as 1 and 0, respectively) and sexual identity.

Two studies reported associations between male identity and IPV victimisation (Finneran & Stephenson, 2014; Turell et al., 2018). A study by Turell et al. (2018) on a non-representative sample found male bisexual-identifying participants reported higher rates of IPV victimisation than their female counterparts. Finneran and Stephenson (2014) reported that physical violence was the most common form of IPV experienced by gay and bisexual men in their study, with more than 8% reporting victimisation from a male partner in the past year. Prevalence of victimisation from female partners for comparison was not provided.

#### Non-Monogamy

Involvement in relationships that were identified as open or polyamorous was found to be associated with IPV victimisation and perpetration in two studies (Head & Milton, 2014; Turell et al., 2018). Findings of Turell et al. (2018) suggested that being in an open relationship was associated with perpetration of IPV, and that perceived infidelity in the relationship was associated with both victimisation and perpetration. A qualitative study by Head and Milton (2014) reported that for some women participants, being engaged in either an open or closed polyamorous relationship could be a means by which male partners exerted domination and control. For example, some reported only being allowed to see other women within an open relationship, or being pressured or coerced to enter into an open relationship.

#### Community Support

Two studies (Friedman et al., 2019; Turell et al., 2018) reported findings in regard to the relationship between gay/bisexual community involvement and IPV. One study found that Black bisexual-identifying men were less likely to disclose their sexual identity to others, which resulted in less gay community support. The relationship between bisexuality and psychosocial morbidity, including IPV, was mediated by the lack of gay community support (Friedman et al., 2019). In contrast, findings of Turell et al. (2018) suggested that involvement in the bisexual community may actually confer risk, with data from that study indicating a positive association between community involvement and IPV victimisation.

#### Previous Experiences of Abuse and Maltreatment

Two studies (Head & Milton, 2014; Taylor & Neppl, 2020) explored relationships between childhood adverse experiences and later IPV victimisation or perpetration among bisexual people. Taylor and Neppl (2020) found that experiencing interparental conflict, harsh parenting or microaggressions throughout childhood was associated with later psychological IPV victimisation and perpetration in an LGBTQ sample. This relationship was moderated by bisexual identity. Qualitative findings from Head and Milton (2014) suggested that growing up in an abusive childhood environment may lead some victims to be more accepting of abuse in their adult relationships. However, other participants in the same study felt that their past experiences of being in an abusive relationship later served as a protective factor by helping them to develop a greater awareness and ability to identify abusive behaviours in new relationships.

#### Inequalities and Discrimination

Some evidence from the present review suggests that societal inequalities and discrimination may contribute to IPV victimisation and perpetration. In a longitudinal analysis, Martin-Storey and Fromme (2021) reported that discrimination experiences mediated the relationship between bisexual identity and dating violence. Additionally, Turell et al. (2018) found that Black/African American participants reported higher IPV victimisation and perpetration scores than other racial identities included in the analysis. Similarly, Pittman et al. (2020) suggest that Black bisexual women who slept with a male in the past year were marginally more likely to experience IPV than other bisexual women. Findings from a study by Finneran and Stephenson (2014), indicated that minority stress from experiencing discrimination was associated with increased risk of experiencing IPV among all participants. Using structural equation modelling, Taylor and Neppl (2020) found that experiencing microaggressions (words and behaviours from others that communicate hostility) was associated with increased IPV victimisation and perpetration, a relationship that was moderated by bisexual identity. Bisexual-identifying men in the study by Finneran and Stephenson (2014) reported experiencing higher rates of racial discrimination than homosexual men, and experiences of racial discrimination were found to be associated with increased odds of sexual IPV victimisation. Seemingly contrasting with findings from these studies, Coston's (2017) findings suggested that cumulative social power (based on a composite measure of age, race/ethnicity, education, income, immigration status or indigeneity) increases bisexual women’s likelihood of experiencing sexual, emotional, and psychological violence, as well as intimate stalking.

Qualitative data from Head and Milton (2014) illuminate other societal factors that impacted continued IPV victimisation of participants. For example, some participants described feeling that the absence of representations of healthy bisexual relationships in media, popular culture and society hindered their ability to recognise abuse and seek support. Some participants also reported staying in abusive relationships out of fear that acknowledging the abuse would contribute to society’s negative stereotypes about bisexual individuals and relationships.

## Discussion

This review aimed to provide a comprehensive assessment of risk and protective factors for IPV perpetration and victimisation against bisexual victims. Literature on the topic was scarce. Few articles examined IPV risk and protective factors specifically for bisexual-identifying individuals. Of those that did, the topic was often explored as a small part of a broader study across sexual identities or other health outcomes. Drawing firm conclusions about risk and protective factors for IPV against bisexual-identifying victims was limited by the cross-sectional design of most of the included studies. Of the nine studies that met criteria for inclusion, only one was longitudinal (Martin-Storey & Fromme, 2021). This means that the directionality of the associations between most of the variables under study in the included articles cannot be determined. For example, the results of the present review suggest that biphobia is associated with IPV. However, it appears that partner biphobia can act not only as a risk factor for victimisation, but also as an abuse tactic in the victimisation of bisexual individuals.

Nevertheless, there were indications of potentially sexuality-specific factors affecting the risk of IPV among bisexual individuals. The results of this review suggest that identification with the label of bisexuality may confer a greater level of risk than that experienced by those who have sexual relationships with both men and women but who do not self-identify as bisexual. This review, and previous findings such as those of Bermea et al. (2018), suggest some reasons why this might be the case, that is, social isolation due to rejection experienced from both heterosexual and gay communities, as well as harm caused by discriminatory stereotypes of bisexual people as promiscuous and unfaithful. It may be useful for future research to examine differences in risk and protective factors between behaviourally bisexual people and self-identified bisexuals, to further develop understanding of how the bisexual label might act to confer unique risk.

Findings from this review also suggest a relationship between minority stress and IPV. Multiple factors contribute to minority stress, including internalised homophobia, sexuality-based discrimination, and racism (Meyer, 1995). Several included studies found that both internalised homophobia and biphobic discrimination may contribute to an increased risk of IPV victimisation and perpetration. These findings are consistent with the broader literature on the LGBTQ+ community (Badenes-Ribera et al., 2019; Carvalho et al., 2011; Stephenson & Finneran, 2016). Some studies in this review also indicate that there may be an increased risk of IPV among bisexual-identifying individuals of colour (Finneran & Stephenson, 2014; Pittman et al., 2020). These findings, as well as those from Stephenson and Finneran (2016) and Reuter et al. (2017), suggest that additional discrimination based on an individual’s racial identity may increase minority stress and lead to higher risk of IPV. The seemingly contradictory findings described by Coston (2017) warrant further examination. It may be the case that Coston’s (2017) findings may be explained by higher rates of IPV reporting among women with fewer barriers (i.e. less stigma, better access to support, etc.). The finding that greater social power increases risk of victimisation could also represent cases of retribution from partners attempting to restore a power imbalance (Decker et al., 2018). It is also worth noting that while Stephenson and Finneran (2016) and Reuter et al. (2017) specifically measured experiences of racial discrimination, Coston (2017) only measured ethnic minority status as part of an indicator of social power, which may have played a role in producing the discrepant findings.

The findings from the present review in relation to gender dynamics are largely consistent with the wider literature on IPV (Krug et al., 2002), with several articles suggesting an increased risk of victimisation of bisexual women if their partners were male. Fewer articles examined associations between gender and IPV victimisation among bisexual men. Intimate partner violence against men by same or opposite-sex partners, while less commonly reported, remains an important issue. This is especially true given that Turell et al. (2018) reported male bisexual-identifying individuals had higher total victimisation scores than females. However, it should be noted that the findings of Turell et al. (2018) are based on a non-representative sample, so larger scale studies using valid measures of IPV are needed.

Some studies included in this review identified associations between being in a non-monogamous relationship and risk for IPV victimisation and perpetration. Mixed evidence exists regarding whether bisexual individuals embrace non-monogamy at higher rates than other groups (Anderson et al., 2015; Haupert et al., 2016; Weinberg et al., 1994). However, it is important to once again emphasise that the cross-sectional and preliminary nature of the findings from the studies included in the present review preclude inferences about directionality of the relationships between these variables. This is particularly relevant in light of the findings of Head and Milton (2014), which showed how perpetrators of IPV may coerce victims to enter into polyamorous relationships; it follows that an association between these variables could therefore represent a manifestation of the abuse itself, rather than a risk factor as such.

Seemingly contradictory results emerged in the included studies regarding the role of gay/bisexual community involvement. The finding that lack of gay community support may be associated with increased risk of experiencing IPV is supported by Meyer (2003) minority stress theory, which suggests that community involvement may protect against adverse outcomes. Bisexual individuals face marginalisation from the heterosexual community as well within the gay/lesbian community; this lack of community belongingness and social support may increase minority stress (Turell et al., 2012) and in turn, vulnerability to IPV. Turell et al. (2018) suggest that their finding that bisexual community involvement increased risk of IPV victimisation may be due to increased jealousy from access to more potential partners. Perhaps the finding may also have a relationship with the apparent risk associated with self-identification with the bisexual label – it could be that the finding from Turell et al., (2018) is capturing some element of this dynamic, rather than the involvement with the community per se. An alternative explanation may be related to the size of the bisexual or LGBTQ+ community. Bornstein et al. (2006) suggest that in areas where the community is small, it is likely that the victim and perpetrator share friends. A small social circle may enable abusers to isolate their partners from their friends, as well as prevent individuals from seeking support within the community by negatively influencing the perceptions of mutual friends (Bornstein et al., 2006; Duke & Davidson, 2009).

### Implications

#### Implications for Research

In addition to examining several risk and protective factors for IPV among bisexual individuals, this review highlights the scarcity of literature on this topic. Given that bisexual individuals appear to be at an increased risk of experiencing IPV compared to their monosexual counterparts (Bermea et al., 2018; Messinger, 2011; Walters et al., 2013), further research specific to this population is needed. For example, research examining the mechanisms of the relationship between bisexual identity and IPV would be useful. Substance use is a well-documented risk factor for both IPV perpetration and victimisation among the wider population (Cafferky et al., 2018; Fals-Stewart et al., 2003; Lipsky et al., 2005; Yu et al., 2019). However, there are currently no studies examining this relationship specifically among bisexual-identifying individuals. Empirical evidence on this relationship is required, particularly considering that bisexual individuals may be at greater risk for substance abuse than other sexual orientations (Green & Feinstein, 2012; Kerr et al., 2014; Ross et al., 2014). Literature also suggests that sexual minority individuals experience higher rates of child abuse and neglect than their heterosexual counterparts (Alvy et al., 2013; Austin et al., 2008; Zou & Andersen, 2015). Two studies in this review identified adverse childhood experiences as risk factors for IPV perpetration and victimisation against bisexual individuals (Head & Milton, 2014; Taylor & Neppl, 2020), and literature suggests that adverse childhood experiences are among the most common predictors of IPV more generally (Costa et al., 2015; Jung et al., 2019). Thus, more research is needed to determine the nature of the relationship between adverse childhood experiences and IPV among bisexual individuals. Longitudinal research is also needed to ascertain the direction of the associations between IPV and factors such as non-monogamy. Given the contrasting findings of Goldberg and Meyer (2013) and Turell et al. (2018) in relation to community involvement, more studies are needed to better understand this relationship and how social support may be harnessed to produce protective effects.

The preliminary quality analysis highlighted limitations of the existing literature on IPV against bisexual victims. The need for more longitudinal research has already been discussed. Additionally, problems with the measurement of the construct of IPV is a well-documented issue affecting the validity of IPV research generally (Walby et al., 2017); this issue was once again identified as a factor impacting interpretability of the findings of the present review. Included studies measured IPV using various definitions and approaches – a combination of standardised scales and simple dichotomous measures. Use of more consistent definitions and measures of IPV across studies would improve comparability and aid the assessment of the relative impact of various risk and protective factors, and of the effectiveness of preventative interventions. Additionally, findings from the included literature lack generalisability across contexts. Eight studies were based in the United States (Coston, 2017; Finneran & Stephenson, 2014; Friedman et al., 2019; Goldberg & Meyer, 2013; Martin-Storey & Fromme, 2021; Pittman et al., 2020; Taylor & Neppl, 2020; Turell et al., 2018), and one in the United Kingdom (Head & Milton, 2014). As cultural norms and acceptance of bisexuality vary both within and across countries, it is likely that risk factors for IPV are also influenced by these social and cultural contexts. These nuances are not captured within the current review, and more contextually and culturally diverse research is therefore needed.

#### Implications for Policy and Practice

This review provides some insight as to how services and policies can better support bisexual individuals. For example, the apparent relationship between minority stress and IPV suggests a clear need for victim services that are equipped to deliver supports that acknowledge the specifics of the experience of IPV in bisexual people. Service providers should also be aware of factors such as the potential that bisexual victims may encounter specific challenges related to accessing social supports. Delivering services that meet the needs of bisexual victims may require the development of more specialist services for communities most affected by minority stress, as well as greater education and sensitisation to minority stress issues within mainstream services. Doing so may help to increase visibility, accessibility and uptake of support services by the bisexual community. Service providers should avoid assuming the sexual orientation of victims based on the sex of their current partner, in order to prevent missing sexuality-specific forms violence or risk factors.

Prevention programmes, including perpetrator programmes, should also include consideration of the particularities of bisexual people’s experiences of IPV, especially with respect to the ways in which domination and control may be exerted based on biphobic stereotypes. The results of the present review suggest that partner biphobia could be highlighted in violence prevention awareness campaigns as a potential early warning sign of abuse. Educational initiatives and awareness campaigns may also play a role in supporting bi-visibility and combatting negative stereotypes. In relation to perpetrator programmes, evidence suggests that adapting interventions to perpetrator risk, criminogenic need and treatment responsivity may improve effectiveness (Andrews & Bonta, 2010; Travers et al., 2021). Data from the present review suggests a basis for investigating internalised homophobia as a potential treatment target for LGBTQ+ perpetrators within such a framework. In general, rigorously designed intervention studies testing perpetrator programmes and other preventive interventions to prevent IPV against bisexual victims is an important priority for future research.

### Limitations

Although the present review has provided a comprehensive assessment of risk and protective factors for IPV perpetrated against bisexual victims, some limitations must be noted. First, limiting inclusion to only studies written in English may have excluded literature from more diverse contexts. Additionally, this review only included literature with bisexual-identifying participants. In doing so, consideration of behaviourally bisexual individuals, who may not identify with the bisexual label, was omitted. Future research should examine IPV experiences among both bisexual-identifying and behaviourally bisexual individuals, as it is likely that the issues facing each group may differ. Analysing both groups separately will help capture nuances and issues distinct to each group.

## Conclusion

The evidence analysing risk factors for IPV in general has historically been significantly under-developed in comparison to the evidence for risk of engaging in general criminality (Andrews & Bonta, 2010). This is especially true of risk factors pertaining to IPV in marginalised groups. This systematic scoping review has analysed the existing literature on risk and protective factors for IPV perpetrated against bisexual individuals, a population that is vastly under-represented within the current literature, despite experiencing a disproportionate risk of IPV. The review identified a number of risk factors, such as bisexual identity, internalised homophobia, discrimination, partner gender, negative childhood experiences and non-monogamy, among others. However, several gaps in the evidence were identified. There was limited focus on protective factors within the included studies. Future studies examining potential protective factors, such as social support, will be needed to inform service design. Additionally, among the few studies that were identified, only one was longitudinal in design. More longitudinally designed research is needed to infer causality between identified risk factors and IPV victimisation and perpetration. A clearer understanding of causality between variables will help to better inform both IPV prevention efforts as well as service provision for bisexual populations.
